# Diagnostic accuracy of adding copeptin to cardiac troponin for non-ST-elevation myocardial infarction: A systematic review and meta-analysis

**DOI:** 10.1371/journal.pone.0200379

**Published:** 2018-07-06

**Authors:** Hyungoo Shin, Bo-Hyoung Jang, Tae Ho Lim, Juncheol Lee, Wonhee Kim, Youngsuk Cho, Chiwon Ahn, Kyu-Sun Choi

**Affiliations:** 1 Department of Emergency Medicine, College of Medicine, Hanyang University, Seoul, Korea; 2 Department of Preventive Medicine, College of Korean Medicine, Kyung Hee University, Seoul, Korea; 3 Convergence Technology Center for Disaster Preparedness, Hanyang University, Seoul, Korea; 4 Department of Emergency Medicine, College of Medicine, Hallym University, Seoul, Korea; 5 Department of Biomedical Engineering, Graduate School of Medicine, Hanyang University, Seoul, Korea; 6 Department of Emergency Medicine, Armed Forces Yangju Hospital, Yangju, Korea; 7 Department of Neurosurgery, College of Medicine, Hanyang University, Seoul, Korea; University of Palermo, ITALY

## Abstract

**Introduction:**

This study aimed to determine the diagnostic accuracy of adding copeptin to cardiac troponin (cTn) on admission to the emergency department (ED) for non-ST elevation myocardial infarction (NSTEMI) compared to cTn alone.

**Materials and methods:**

A literature search of MEDLINE, EMBASE, and the Cochrane Library was performed (search date: April 13, 2018). Primary studies were included if they accurately reported on patients with symptoms suggestive of acute myocardial infarction and measured both cTn alone and cTn with copeptin upon admission to the ED. The patients with evidence of ST elevation myocardial infarction were excluded. To assess the risk of bias for the included studies, the QUADAS-2 tool was used.

**Results:**

The study participants included a total of 7,998 patients from 14 observational studies. The addition of copeptin to cTn significantly improved the sensitivity (0.81 [0.74 to 0.87] vs. 0.92 [0.89 to 0.95], respectively, p <0.001) and negative predictive value (0.96 [0.95 to 0.98] vs. 0.98 [0.96 to 0.99], respectively, p <0.001) at the expense of lower specificity (0.88 [0.80 to 0.97] vs. 0.57 [0.49 to 0.65], respectively, p <0.001) compared to cTn alone. Furthermore, adding copeptin to cTn showed significantly lower diagnostic accuracy for NSTEMI compared to cTn alone (0.91[0.90 to 0.92] vs. 0.85 [0.83 to 0.86], respectively, p < 0.001).

**Conclusions:**

Adding copeptin to cTn improved the sensitivity and negative predictive value for the diagnosis of NSTEMI compared to cTn alone. Thus, adding copeptin to cTn might help to screen NSTEMI early upon admission to the ED.

## Introduction

Acute myocardial infarction (AMI) is the leading cause of death and disability worldwide [1]. A 12-lead electrocardiography (ECG) recording, biomarker analysis, and clinical assessment are commonly performed for the initial evaluation of AMI [2]. While ST elevation myocardial infarction (STEMI) can be readily identified through a clinical assessment and ECG, the diagnosis of non-ST elevation myocardial infarction (NSTEMI) is made based on the serum biomarkers of myocardial necrosis [3].

Cardiac troponin (cTn) has been regarded as a standard marker for myocardial injury, and its elevation is a component of the universal definition of AMI [1]. However, cTn is not sufficiently sensitive within the first hours of myocardial injury, a phenomenon called the “troponin-blind” period [4]. Although high-sensitivity cTn assays are increasingly being used to allow for a more rapid assessment, alternative biomarkers that may be more sensitive to early myocardial injury have gained increasing interest.

Recently, copeptin, an acute endogenous stress neuropeptide [5], has gained attention in the clinical field, with results available within 60 min [6,7]. The combination of copeptin and cTn has been proposed to be used for the assessment of patients with suspected AMI [8,9]. Although copeptin is nonspecific to myocardial injury, it responds to an immediate neural trigger with concentrations rising early and decreasing gradually over several hours [10]. This rapid release may help to cover the “troponin-blind” period.

Hence, a diagnostic systematic review and meta-analysis were performed to determine the diagnostic accuracy of examining both copeptin and cTn in identifying NSTEMI when compared to cTn alone on admission to the emergency department (ED).

## Materials and methods

### 1. Study design

This study was conducted in accordance with the principles outlined by the Systematic Reviews of Diagnostic Test Accuracy [11] and the Preferred Reporting Items for Systematic Reviews and Meta-analysis (PRISMA) groups [12].

### 2. Eligibility criteria

#### 2.1. Type of studies

Relevant studies were included in our meta-analysis if they: (1) reported results from patients with symptoms suggestive of AMI without evidence of ST-segment elevation, (2) measured both cTn alone and combination of cTn and copeptin on admission to the ED, or (3) assessed the diagnostic performance. Studies that (1) included patients with STEMI, (2) had insufficient data in spite of contacting the authors, or (3) did not meet the criteria for enrollment in our study were excluded.

#### 2.2. Participants

Our selected studies included adult patients who presented to the ED with suspected AMI using both cTn alone and combination of cTn and copeptin.

#### 2.3. Index tests

This study included only studies examining the diagnostic accuracy of both baseline cTn alone and adding copeptin to cTn measured in blood samples obtained upon admission to the ED. The cTn index tests included conventional and high-sensitivity assays. Copeptin index tests included manual immunoluminometric assays and automated immunofluorescent assays.

#### 2.4. Reference tests

The reference standard was comprised of all available medical records including cTn assay results. NSTEMI is defined by electrocardiographic ST-segment depression or prominent T-wave inversion and positive biomarkers in the absence of ST-segment elevation and in a clinical assessment [1,13].

### 3. Search strategy

Two experienced reviewers (H. Shin and C. Ahn) performed the literature search on April 13, 2018. The search encompassed the MEDLINE (1974 to April 11, 2018) and EMBASE (1974 to April 11, 2018) databases via the Ovid interface and the Cochrane Library (all years). The following keywords were searched: copeptin, myocardial infarction, acute coronary syndrome, coronary artery disease, and angina. No language restrictions and no methodology filters were used. S1 Table presents the details of the search strategies. Articles that reported any prospective or retrospective observational studies were included.

### 4. Study selection

**Fig 1:**
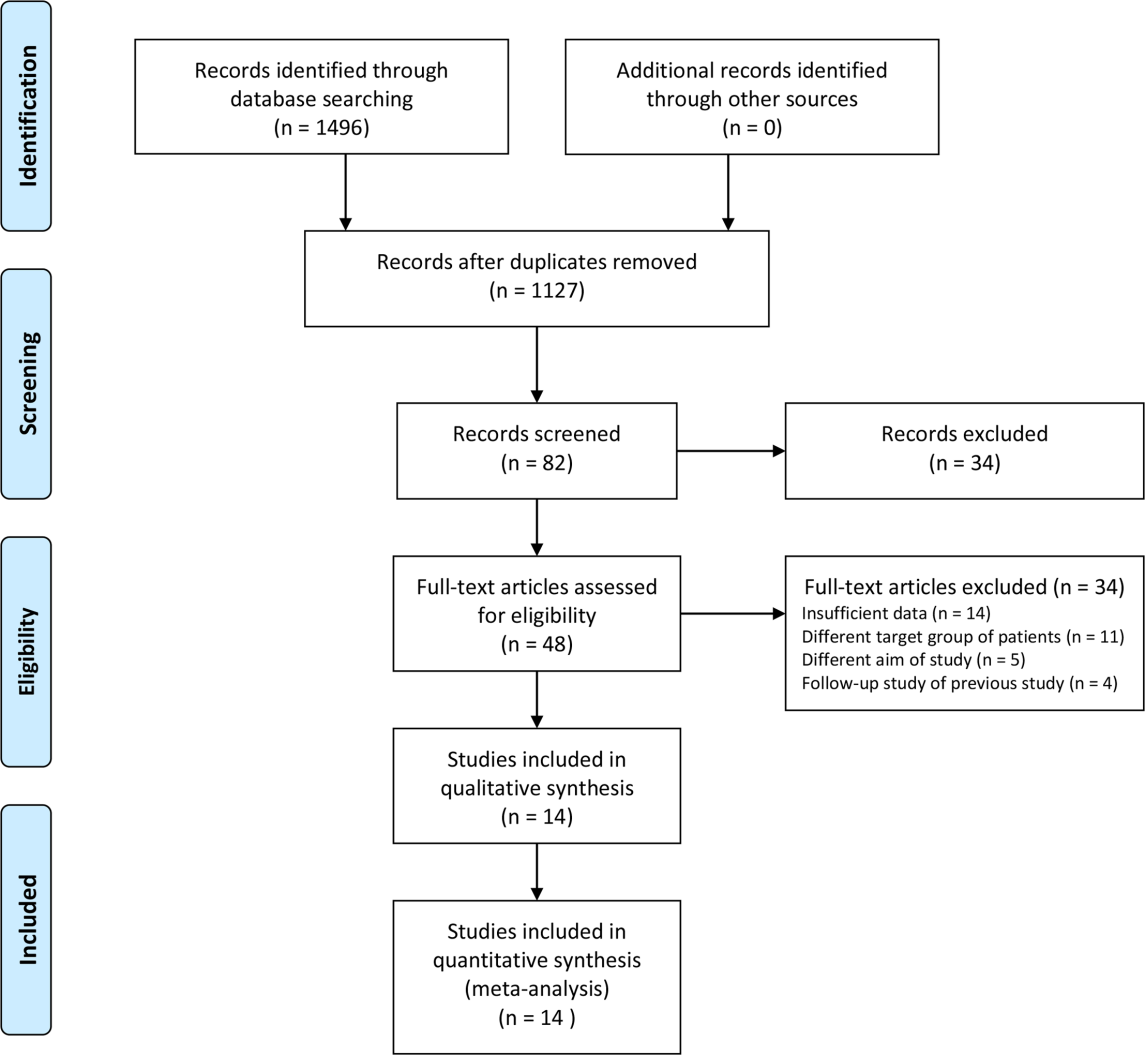
Flow diagram of study selection.

The reference management software Endnote 7.4 was used for all identified studies. The title, abstract, and type of each of the identified articles were examined by two reviewers. Those articles that fell under the exclusion criteria (reviews, case reports, editorials, letters, comments, conference abstracts, or meta-analyses; animal studies; duplicate studies; irrelevant population; irrelevant index test; and irrelevant outcomes (Fig 1)) were not considered. In case of disagreement between the two reviewers, a third reviewer (BH Jang) intervened, and differences were discussed until a consensus was reached. The full texts of the chosen articles were acquired, which were then rescreened and evaluated more thoroughly for eligibility using the same exclusion criteria.

### 5. Data extraction

The two reviewers obtained the characteristics and results of selected studies. Studies with lacking data despite contacting the authors were excluded from the meta-analysis. Data regarding true-positive, false-positive, false-negative, and true-negative results for individual studies were obtained. Variables such as the use of different cut-points for copeptin and both conventional and high-sensitivity cTn were considered. The following variables were also extracted from studies: first author, year of publication, country, study population, inclusion period, assay method for cTn and copeptin detection, and patients’ baseline characteristics. The corresponding author (TH Lim) had full access to all the data in the study and took responsibility for its integrity and the data analysis.

### 6. Assessment of methodological quality

Two reviewers assessed the methodological quality of the primary studies at the study level. Patient selection, index test, reference standard, flow, and timing were assessed using a checklist adapted from the Quality Assessment of Diagnostic Accuracy Studies (QUADAS)-2 tool [14]. The key methodological issue of including studies would be the potential for incorporation bias. Elevation of cTn, which is a part of NSTEMI, was included as one of the index tests in this study.

### 7. Statistical analysis

For each primary study, sensitivity and specificity point estimates and corresponding 95% confidence intervals (CI) were calculated from extracted data for cTn alone and cTn with copeptin. We used SAS software, version 9.4 (SAS Institute, Cary, NC, USA), to perform a bivariate random effects model, R version 3.2.3 (r-project.org) with “mada” package, and Review Manager (RevMan) 5 version 5.1.7. The statistical significance for hypothesis testing was set at 0.05 for 2-tailed heterogeneity testing and at 0.10 for 2-tailed tests. Dichotomous variables are reported as proportions (%), whereas continuous variables are reported as mean (standard deviation [SD]) or median (interquartile range [IQR]).

#### 7.1. Summary diagnostic accuracy estimates

The summary estimates of sensitivity, specificity, and positive and negative likelihood ratios were derived from bivariate mixed-effect regression model parameter estimates. The area under the summary receiver operating characteristic curve (AUC) was plotted using logistic estimates of sensitivity and specificity and the respective variance and covariance. True-positive, true-negative, false-positive, and false-negative rates were used to compute the sensitivity, specificity, positive predictive value (PPV), and negative predictive value (NPV).

#### 7.2. Sensitivity analyses

A sensitivity analysis was performed for all studies except for one study where the enrolled patients were aged ≥70 years [15]. Subgroup analysis was also performed for the studies comparing the addition of copeptin to cardiac troponin I (cTnI) and high-sensitivity cardiac troponin T (hs-cTnT) assays.

## Results

### 1. Characteristics of study subjects

#### 1.1. Literature search

A total of 1,496 records were identified through database searching (Fig 1). After removing 369 duplicates, the titles and abstracts for 1,127 records were screened for eligibility. Of these, 48 records were identified as being potentially relevant, and full-text articles were retrieved for a more thorough review. After excluding 34 manuscripts after assessment of the full-text articles, 14 studies, which enrolled 7,998 patients, were included in the meta-analysis.

#### 1.2. Characteristics of included studies

The 14 studies included a total of 7,998 patients, and the prevalence of NSTEMI was 14.2% (range 6.0–35.6%) [10,15–27]. Only the CHOPIN study was a multinational study conducted in the USA and Europe, whereas all the other studies were conducted in Europe. The diagnostic threshold for copeptin was 14 pmol/L (range 7.4–14 pmol/L) in seven studies, and the cTn index tests consisted of cTnI assays in six studies (range 40–100 ng/L) and hs-cTnT assays in eight studies with a diagnostic threshold of 14 ng/L (Table 1). S2 Table presents the patients’ baseline characteristics. The number of true-positive, false-positive, false-negative, and true-negative values with the corresponding sensitivities, specificities, PPV, and NPV for NSTEMI is provided based on the cut-points for cTn (S3 Table) and the addition of copeptin to cTn (S4 Table).

**Table 1:** Study characteristics.

Author	Year published	Inclusion period and country	Patients (n)	NSTEMI (% of total)	Assay cut-off value		
					cTnI (ng/L)	Hs-cTnT (ng/L)	Copeptin (pmol/L)
Alquézar [16]	2017	2009.5–2010.6	297	63 (21.2)		Roche	Ultrasensitive copeptin KRYPTOR
		Espana				(14)	(10)
Bahrmann [15]	2013	2011.1–2011.9	306	38 (12.4)		Roche	Ultrasensitive copeptin KRYPTOR
		Germany				(14)	(14)
Charpentier [17]	2012	2006.3–2007.3	641	95 (14.8)	Siemens Healthcare		Copeptin KRYPTOR
		France			(100)		(14)
Collinson [18]	2013	2007.1–2008.6	803	63 (7.8)		Roche	Copeptin KRYPTOR
		UK				(14)	(7.4)
Dupuy [19]	2012	2009.12–2010.4	121	15 (12.4)	Access2 analyzer		Copeptin KRYPTOR
		France			(40)		(10.4)
Eggers [20]	2012	2002.10–2003.8 (FASTER I)	360	128 (35.6)		Roche	Ultrasensitive copeptin KRYPTOR
		2000.5–2001.3 (FASTER II)				(14)	(14)
		Sweden					
Jacobs [21]	2015	2010.9–2011.5	584	95 (16.3)	Siemens Healthcare		Copeptin KRYPTOR
		Netherlands			(45)		(14)
Maisel (CHOPIN) [10]	2013	NR	1927	116 (5.9)	Siemens Ultra		Copeptin KRYPTOR
		16 study centers; USA, Switzerland, Germany			(40)		(14)
Meune [22]	2011	2009.6–2009.11	58	13 (22.4)		Roche	Copeptin KRYPTOR
		France				(14)	(14)
Ricci (COPACS) [23]	2016	2013.6–2013.12	196	29 (14.8)	Siemens medium sensitive		Ultrasensitive copeptin KRYPTOR
		Italy			(45)		(10)
Sebbane [24]	2013	2009.12–2011.11	167	25 (15.0)		Roche	Ultrasensitive copeptin KRYPTOR
		France				(14)	(13.11)
Thelin [25]	2013	2011.3–2011.7	478	70 (14.6)		Roche	Copeptin KRYPTOR
		Sweden				(14)	(14)
Vafaie [26]	2015	2010.8–2011.11	131	28 (21.4)		Roche	Ultrasensitive copeptin KRYPTOR
		Germany				(14)	(10)
Wildi (APACE) [27]	2015	2006.4–2012.9	1929	358 (18.6)	Siemens Ultra		Copeptin LUMItest
		Europe			(0.04 uL/L)		(9)

Abbreviations: NSTEMI = non-ST-elevation myocardial infarction; cTnI = cardiac troponin I; hs-cTnT = high-sensitivity cardiac troponin T; NR = not reported.

#### 1.3. Assessment of study quality

All included studies were assessed to determine if they were low risk in patient selection and applicability (S1 Fig). Eight studies were considered low risk. One study was assessed as high risk for flow and timing bias. In addition, the risk of bias for the index test and reference test were unclear in four and five studies, respectively.

### 2. Main results

#### 2.1. Comparison of overall diagnostic accuracy for cardiac troponin alone and the addition of copeptin

**Fig 2:**
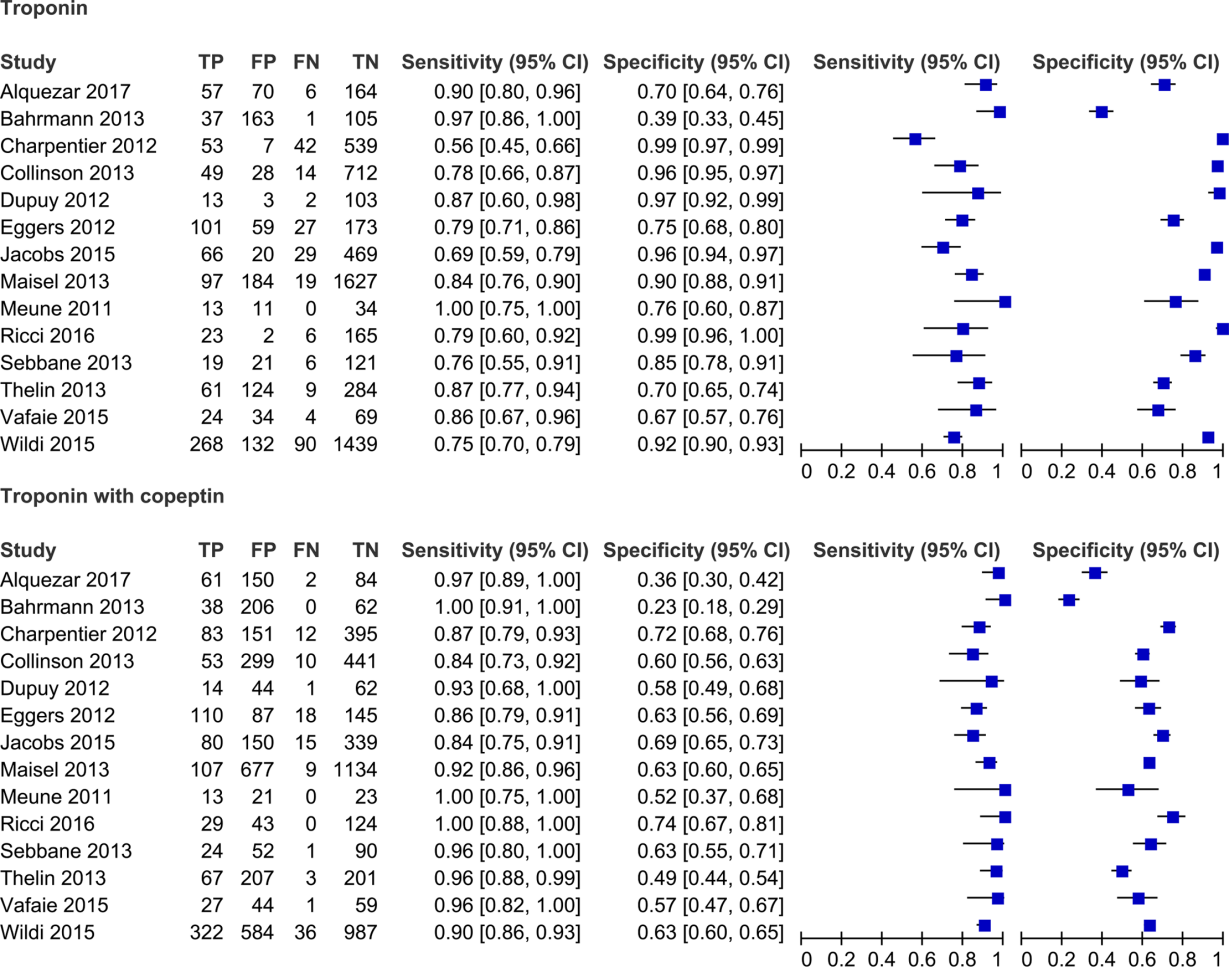
Coupled forest plot for the combined assessment of cardiac troponin and copeptin for NSTEMI.

**Fig 3:**
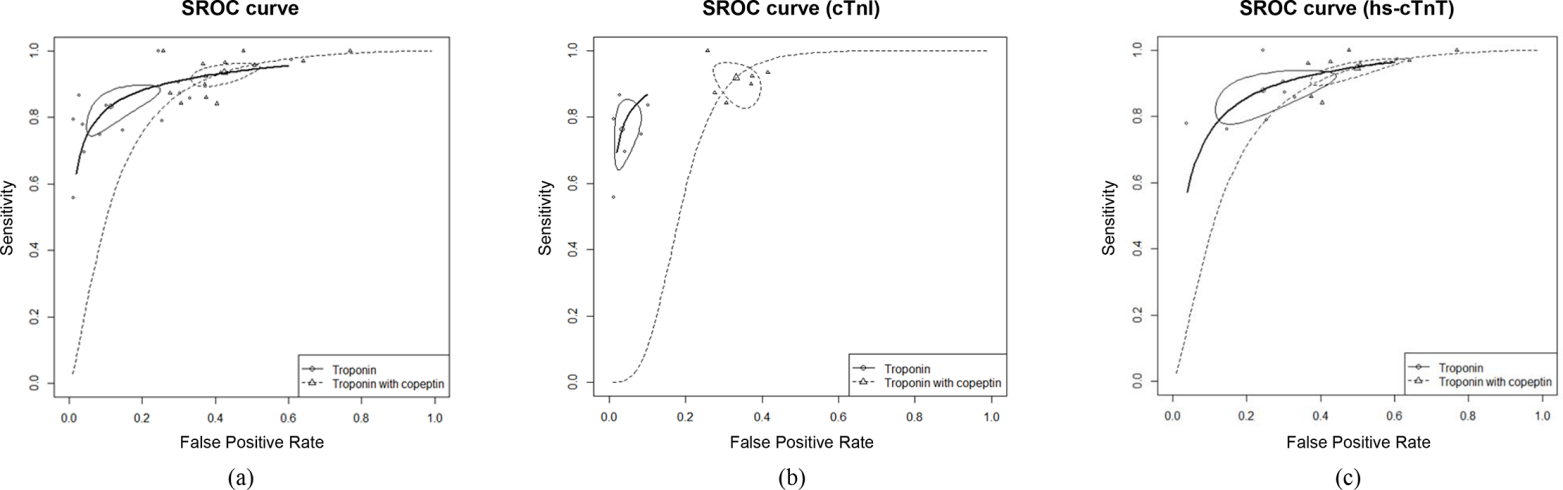
Summary receiver operating characteristic curves for the assessment of cardiac troponin alone and cardiac troponin with copeptin for identifying of non-ST elevation myocardial infarction (14 studies). (a) The pooled area under the curve for the assessment of cardiac troponin alone and cardiac troponin with copeptin are 0.91 and 0.85, respectively (p < 0.001). (b) For studies that assessed cardiac troponin I, the area under the curve scores for the assessment of cardiac troponin I alone and cardiac troponin I with copeptin are 0.93 and 0.80, respectively (p < 0.001). (c) When limited to studies assessing high-sensitivity cardiac troponin T, the area under the curve scores for the assessment of high-sensitivity cardiac troponin T alone and high-sensitivity cardiac troponin T with copeptin are 0.90 and 0.83, respectively, p < 0.001.

The combined assessment of cTn and copeptin ranged from 0.84 to 1.00 for sensitivity and from 0.23 to 0.74 for specificity (Fig 2). On the other hand, cTn alone showed a sensitivity ranging from 0.56 to 1.00 with specificity ranging from 0.39 to 0.99. Overall, the addition of copeptin to cTn significantly improved the sensitivity (0.81 vs. 0.92, p < 0.001) and NPV (0.96 vs. 0.98, p < 0.001) and decreased the specificity (0.88 vs. 0.57, p < 0.001) compared to cTn alone (Table 2). In addition, adding copeptin to cTn has a lower diagnostic accuracy (0.91 vs. 0.85, p < 0.001) than the cTn alone (Fig 3).

**Table 2:** Paired comparison of diagnostic accuracy for adding copeptin to cardiac troponin for NSTEMI*.

Overall (95% CI)	cTn	cTn with copeptin	difference	*p-value*
Pooled sensitivity	0.81 (0.74, 0.87)	0.92 (0.89, 0.95)	0.11 (0.08, 0.14)	< .001
Pooled specificity	0.88 (0.80, 0.97)	0.57 (0.49, 0.65)	-0.31 (-0.35, -0.27)	< .001
Pooled PPV	0.58 (0.45, 0.71)	0.28 (0.20, 0.37)	-0.30 (-0.36, -0.24)	< .001
Pooled NPV	0.96 (0.95, 0.98)	0.98 (0.96, 0.99)	0.02 (0.01, 0.03)	< .001
Pooled AUC	0.91 (0.90, 0.92)	0.85 (0.83, 0.86)	NA	< .001

*The prevalence of target condition was 14.2% for the cohort of patients with suspicion of NSTEMI

Abbreviations: 95% CI = 95% confident interval; cTn = cardiac troponin; PPV = positive predictive value; NPV = negative predictive value; AUC = area under the summary receiver operating characteristic curve; NA = not available.

#### 2.2. Subgroup analysis for adding copeptin between cardiac troponin I and high-sensitivity cardiac troponin T

The addition of copeptin to either cTnI or hs-cTnT significantly improved the sensitivity and decreased the specificity compared to cTnI or hs-cTnT alone (Table 3). More specifically, adding copeptin increased the sensitivity for cTnI (0.71 vs. 0.89, p < 0.001) and hs-cTnT (0.86 vs. 0.93, p < 0.001) and reduced the specificity for cTnI (0.96 vs. 0.67, p < 0.001) and hs-cTnT (0.76 vs. 0.50, p < 0.001). Adding copeptin increased the NPV for cTnI (0.96 vs. 0.97, p = 0.011), but adding copeptin decreased the NPV for hs-cTnT (0.97 vs. 0.94, p = 0.001). Adding copeptin had a lower diagnostic accuracy compared to both cTnI and hs-cTnT.

**Table 3:** Subgroup analysis for assessing the diagnostic accuracy of adding copeptin to cardiac troponin.

Type of cTn	cTnI				hs-cTnT			
No. of studies	6				8			
No. of patients*	5398				2600			
Diagnostic tests (95% CI)	cTnI	cTnI with copeptin	Difference	*p-value*	hs-cTnT	hs-cTnT with copeptin	Difference	*p-value*
Sensitivity	0.71 (0.60, 0.82)	0.89 (0.86, 0.93)	0.18 (0.14, 0.22)	< .001	0.86 (0.79, 0.93)	0.93 (0.91, 0.96)	0.07 (0.04, 0.10)	< .001
Specificity	0.96 (0.92, 1.00)	0.67 (0.61, 0.72)	-0.29 (-0.31, -0.27)	< .001	0.76 (0.60, 0.91)	0.50 (0.40, 0.59)	-0.26 (-0.33, -0.19)	< .001
PPV	0.73 (0.58, 0.89)	0.29 (0.17, 0.40)	-0.44 (-0.50, -0.38)	< .001	0.44 (0.33, 0.56)	0.48 (0.30, 0.65)	0.04 (-0.03, 0.11)	0.136
NPV	0.96 (0.94, 0.98)	0.97 (0.96, 0.99)	0.01 (0, 0.02)	0.011	0.97 (0.95, 0.99)	0.94 (0.89, 0.98)	-0.03 (-0.05, -0.01)	0.001
AUC	0.93 (0.92, 0.95)	0.80 (0.78, 0.82)	NA	< .001	0.90 (0.88, 0.92)	0.83 (0.80, 0.86)	NA	< .001

*The prevalence of target condition was 13.1% for the cohort evaluating cTnI and 16.5% for the cohort evaluating hs-cTnT assay.

Abbreviations: cTn = cardiac troponin; cTnI = cardiac troponin I; hs-cTnT = high sensitivity cardiac troponin T; No = number; 95% CI = 95% confident interval; PPV = positive predictive value; NPV = negative predictive value; AUC = area under the summary receiver operating characteristic curve; NA = not available.

#### 2.3. Sensitivity analysis

The sensitivity analysis was performed in 13 studies, with the exception of one study [15]. Adding copeptin to cTn significantly improved the NPV (0.96 vs. 0.98, p = 0.001) compared to cTn alone (S5 Table). Upon restricting the analysis to the seven studies providing data for hs-cTnT, adding copeptin to cTn significantly decreased the NPV (0.96 vs. 0.93, p = 0.001) compared to cTn alone. As a result of the sensitivity analysis, adding copeptin to cTn showed lower diagnostic accuracy (0.91 vs. 0.83, p < 0.001) compared to cTn alone.

## Discussion

As demonstrated by this meta-analysis, the addition of copeptin significantly increased the sensitivity and NPV of cTn in NSTEMI patients compared to cTn alone. However, adding copeptin to cTn did not improve the diagnostic accuracy of NSTEMI as assessed by the pooled AUC, when compared to cTn alone. Sensitivity analysis was performed on all studies except for one study [15]; adding copeptin to cTn increased NPV but showed lower diagnostic accuracy to cTn alone.

In two recent meta-analyses, adding copeptin to cTn showed a higher sensitivity and lower specificity in the early rule-out of suspected AMI patients [4,28]. The patients with STEMI should be assessed for immediate reperfusion therapy [3]; suspected STEMI patients should not have to wait for laboratory results [1]. Meanwhile, cTn is essential in the diagnosis and management of patients with suspected NSTEMI [2]. It is of diagnostic value when ECG reveals no ST segment elevation in the presence of a high suspicion of myocardial necrosis. Thus, diagnostic characteristics of cardiac biomarkers for NSTEMI patients were assessed in this study.

Myocardial injury triggers neuroendocrine changes that result in the rapid release of copeptin into the circulation [29]. The measurement of copeptin <6 hours from the presentation of chest pain would make best use of its early release kinetics [10,30,31]. In AMI patients, copeptin levels are elevated 0–4 hours after the symptoms occur [32–34]. This rapid-release kinetic can cover the cTn delayed release period known as the “troponin-blind” period.

Physicians are frequently faced with the clinical decision-making scenario to either retain the patient for further observation or discharge low-risk patients whose initial cTn values were negative [35]. The sensitivity and specificity of a test have limited clinical usefulness as they cannot be used to estimate the probability of disease in an individual patient [36]. However, NPV, which tells us the probability of not having a disease, given as a negative test may be more useful to rule out AMI. Clinical assessment and the more reliable high NPV would help to rule out patients highly likely to not have AMI in the ED. Reichlin et al. tried to determine whether a combined testing strategy using copeptin and cTn could result in improved NPV for the rapid ruling-out of suspected AMI patients [37].

In this study, we observed how the NPV varies when adding copeptin to cTn compared to cTn alone; the addition of copeptin to cTn significantly improved NPV compared to cTn alone. The discharge of patients negatively presenting with cTn would result in 4% of patients being inappropriately discharged with NSTEMI. However, adding copeptin to cTn would lead to a 50% reduction of inappropriate discharges. Nevertheless, this suggests that 2% of patients with both initially negative copeptin and negative cTn will still have an AMI. Although the NPV had significantly improved, the slight increase in the NPV raises questions about how effective it is in clinical situations.

The early diagnosis of AMI has significantly improved with the recent development of high-sensitivity assays, which reliably measure cTn concentrations that were not detected by previous generations of tests [38–40]. High-sensitivity cTn assays allow a more frequent and earlier detection of AMI in patients with chest pain than conventional assays [2]. Therefore, we analyzed the diagnostic characteristics of adding copeptin to cTn by distinguishing between cTnI and hs-cTnT.

As demonstrated by this meta-analysis, adding copeptin to either cTnI or hs-cTnT significantly improved the sensitivity and reduced the specificity compared to either cTnI or hs-cTnT alone. Adding copeptin to cTnI significantly improved the NPV compared to cTnI alone, but adding copeptin to hs-cTnT significantly decreased the NPV. The effect of copeptin on the NPV is different when copeptin is combined with hs-cTnT from cTnI; the addition of copeptin could be useful when applied with cTnI. The fact that only hs-cTnT was included in this study should be considered, because it is not obvious that hs-cTnT and hs-cTnI can lead to the same diagnostic accuracy, given that the analytical performances of these assays can differ significantly.

A study by Reinstadler et al. on the usefulness of copeptin in patients with suspected AMI in comparison with routine biomarkers indicated that the advantages of the dual marker strategy appear insignificant when hs-cTnT assays are used [41]. Meanwhile, Potocki et al. showed that when copeptin is used in combination with hs-cTnT, it significantly improved the diagnostic and prognostic accuracy [42]. This study assessed for patients with pre-existing coronary artery disease. These specific patients may show different diagnostic performances of the biomarker. Other studies concluded that the dual strategy should be applied to clinically selected patients with low to intermediate risk of AMI, so as to maximize the NPV if hs-cTnT assays are not available or approved for clinical use [43,44]. Future studies about the diagnostic performance of the adding copeptin to hs-cTnT are necessary.

As shown in the pooled AUC, adding copeptin to cTn showed lower diagnostic accuracy for NSTEMI compared to cTn alone. Emergency physicians involved in the management of older patients encounter the diagnostic challenges of improved sensitivity but decreased specificity of hs-cTnT assays on a daily basis [45]. Reiter et al. revealed that the best cut-off value to rule in AMI varies substantially with age; older patients have nearly four times higher cut-off values with hs-cTnT [46]. As a result of the sensitivity analysis, except for one study that enrolled patients aged ≥70 years [15], adding copeptin to cTn showed lower diagnostic accuracy (0.91 vs. 0.83, p < 0.001) compared to cTn alone, but improved the sensitivity (0.79 vs. 0.91, p < 0.001) and the NPV (0.96 vs. 0.98, p = 0.001). In this setting, the use of copeptin may be helpful in the diagnostic work-up for NSTEMI patients.

This meta-analysis has several important limitations. First, the between-study statistical and clinical heterogeneity was still unresolved in this study. The reasons for heterogeneity in estimates were related to variations of proportions of underling disease and risk factors of AMI, timing of enrollment, copeptin and cTn assays, and the cut-points across the original studies. This may restrict the quality and interpretation of data. Second, a major methodological concern exists in the included studies. There is the potential for incorporation bias with the baseline cTn value serving as the index test and being part of the reference standard of NSTEMI, based on indirect comparison. This may result in overestimating the diagnostic accuracy for cTn and, therefore, decrease the diagnostic value of copeptin. Third, most included studies were performed in Europe. Hence, these findings may not apply to patients from other regions.

## Conclusions

The addition of copeptin to cTn improved the sensitivity and NPV for the diagnosis of NSTEMI compared to cTn alone. Thus, adding copeptin to cTn might help to detect NSTEMI early upon admission to the ED. The addition of copeptin to hs-cTnT could be a useful alternative if hs-cTnT assays are not available or approved for clinical use.

## Supporting information

S1 Table Search strategy. (PDF)

S2 Table Number of true positives, true negatives, false positives, and false negatives based on the cardiac troponin I or high-sensitivity troponin T cut-point for studies providing these data. (PDF)

S3 Table Number of true positives, true negatives, false positives, and false negatives based on the addition of copeptin to cardiac troponin I or high-sensitivity troponin T cut-point for studies providing these data. (PDF)

S4 Table Characteristics of patients in the included studies. (PDF)

S5 Table Sensitivity analysis for diagnostic accuracy of cardiac troponin alone and adding copeptin to cardiac troponin for non-ST elevation myocardial infarction, except for one study that enrolled patients aged ≥70 years. (PDF)

S1 Fig Assessment risk of bias using Quality Assessment of Diagnostic Accuracy Studies—2 tool. (PDF)
