# Supplementary material for: Diagnostic accuracy of adding copeptin to cardiac troponin for non-ST-elevation myocardial infarction: A systematic review and meta-analysis
Source: PLoS One. 2018 Jul 6;13(7):e0200379. doi: 10.1371/journal.pone.0200379 (PMC6034895; doi:10.1371/journal.pone.0200379)
Supplement: S1 Table — (PDF) [file pone.0200379.s001.pdf]

S1 Table. Search strategy

Medline

No.	Searches	Results
1	Myocardial infarction.ti,ab	159864
2	*Infarction/	5482
3	Myocardial infarction/	156910
4	1 or 2 or 3	226130
5	exp Acute Coronary Syndrome/ or exp Coronary Disease/ or exp Coronary Artery Disease/ or exp Myocardial Infarction/ or exp Myocardial Ischemia/	400660
6	exp Angina Pectoris/ or exp Angina, Unstable/ or Angina, Stable/	42098
7	Angina.ti,ab.	49440
8	5 or 6 or 7	411636
9	4 or 8	455731
10	(Glycopeptides or copeptins or "C-terminal provasopressin" or Glycopeptide or copeptin).ab,ti.	10921
11	exp Glycopeptides/	54859
12	(Arg$Vasopressin or "Argipressin" or "Arginine Vasopressin").ab,ti.	10421
13	exp Arginine Vasopressin/	15116
14	10 or 11 or 12 or 13	77975
15	9 and 14	524

Embase

No.	Searches	Results
1	Myocardial infarction.ti,ab.	234668
2	Heart infarction/ or acute heart infarction/ or infarction/ or ST segment elevation myocardial infarction/	355993
3	Myocardial disease/	5432
4	1 or 2 or 3	399488
5	exp heart infarction/	347640
6	exp heart muscle ischemia/	86972
7	exp coronary artery disease/ or exp ischemic heart disease/	731532
8	5 or 6 or 7	731532
9	exp angina pectoris/	93008
10	Angina.ti,ab.	68626
11	9 or 10	109739
12	8 or 11	738240
13	4 or 12	782857
14	(Glycopeptides or copeptins or 'C-terminal provasopressin' or Glycopeptide or copeptin or 'Arg-Vasopressin' or 'Arg Vasopressin' or 'Argipressin' or 'Arginine Vasopressin').ab,ti.	24463
15	exp copeptin/ or exp glycopeptide/ or exp argipressin/	23844
16	14 or 15	33927
17	13 and 16	883

Cochrane library

No.	Searches	Results
1	“myocardial infarction”:ti,ab,kw (Word variations have been searched)	22532
2	MeSH descriptor: [Infarction] explode all trees	20
3	MeSH descriptor: [Myocardial Infarction] explode all trees	10266
4	#1 or #2 or #3	22605
5	MeSH descriptor: [Myocardial Ischemia] explode all trees	25410
6	MeSH descriptor: [Coronary Artery disease] explode all trees	4119
7	MeSH descriptor: [Coronary disease] explode all trees	11982
8	#5 or #6 or #7	25410
9	MeSH descriptor: [Angina Pectoris] explode all trees	4339
10	angina:ti,ab,kw (Word variations have been searched)	10347
11	#9 or #10	10349
12	#8 or #11	29994
13	#4 or #12	38856
14	"copeptin":ti,ab,kw (Word variations have been searched)	102
15	MeSH descriptor: [Glycopeptides] explode all trees or "glycopeptide":ti,ab,kw (Word variations have been searched)	1778
16	MeSH descriptor: [Vasopressins] explode all trees or MeSH descriptor: [Arginine Vasopressin] explode all trees or "vasopressin":ti,ab,kw (Word variations have been searched) or "argipressin":ti,ab,kw (Word variations have been searched)	2174
17	#14 or #15 or #16	3979
19	#13 and #18	89

A literature search was performed on April 13, 2018.
