# Supplementary material for: Diagnostic accuracy of adding copeptin to cardiac troponin for non-ST-elevation myocardial infarction: A systematic review and meta-analysis
Source: PLoS One. 2018 Jul 6;13(7):e0200379. doi: 10.1371/journal.pone.0200379 (PMC6034895; doi:10.1371/journal.pone.0200379)
Supplement: S2 Table — (PDF) [file pone.0200379.s002.pdf]

S2 Table. Number of true positives, true negatives, false positives, and false negatives based on the cardiac troponin I or high-sensitivity troponin T cut-point for studies providing this data.

Study	cTn cut-point (ng/L)	TP (n)	FP (n)	FN (n)	TN (n)	Sensitivity (%)	Specificity (%)	PPV (%)	NPV (%)
Alquezar	hs-cTnT (14)	57	70	6	164	90.5	70.1	44.9	96.5
Bahrmann	hs-cTnT (14)	37	163	1	105	97.4	39.2	18.5	99.1
Charpentier	cTnI (100)	53	7	42	539	55.8	98.7	88.3	92.8
Collinson	hs-cTnT (14)	49	28	14	712	77.8	96.2	63.6	98.1
Dupuy	cTnI (40)	13	3	2	103	86.7	97.2	81.3	98.1
Eggers	hs-cTnT (14)	101	59	27	173	78.9	74.6	63.1	86.5
Jacobs	cTnI (45)	66	20	29	469	69.5	95.9	76.7	94.2
Maisel	cTnI (40)	97	184	19	1627	83.6	89.8	34.5	98.8
Meune	hs-cTnT (14)	13	11	0	34	100	75.6	54.2	100
Ricci	cTnI (45)	23	2	6	165	79.3	98.8	92.0	96.5
Sebbane	hs-cTnT (14)	19	21	6	121	76.0	85.2	47.5	95.3
Thelin	hs-cTnT (14)	61	124	9	284	87.1	69.6	33.0	96.9
Vafaie	hs-cTnT (14)	24	34	4	69	85.7	67.0	41.4	94.5
Wildi	cTnI (40)	268	132	90	1439	74.9	91.6	67.0	94.1

Abbreviations: cTn = cardiac troponin; TP = true positive; FP = false positive; FN = false negative; TN = true negative; NPV = negative predictive value; PPV = positive predictive value; hs-cTnT = high-sensitivity cardiac troponin T; cTnI = cardiac troponin I
