# Supplementary material for: Diagnostic accuracy of adding copeptin to cardiac troponin for non-ST-elevation myocardial infarction: A systematic review and meta-analysis
Source: PLoS One. 2018 Jul 6;13(7):e0200379. doi: 10.1371/journal.pone.0200379 (PMC6034895; doi:10.1371/journal.pone.0200379)
Supplement: S3 Table — (PDF) [file pone.0200379.s003.pdf]

S3 Table. Number of true positives, true negatives, false positives, and false negatives based on the addition of copoptin to cardiac troponin I or high-sensitivity troponin T cut-point for studies providing this data.

Study	Copeptin cut-point (pmol/L)	TP (n)	FP (n)	FN (n)	TN (n)	Sensitivity (%)	Specificity (%)	PPV (%)	NPV (%)
Alquezar	10	61	150	2	84	96.8	35.9	28.9	97.7
Bahrmann	14	38	206	0	62	100	23.1	15.6	100
Charpentier	14	83	151	12	395	87.4	72.3	35.5	97.1
Collinson	7.4	53	299	10	441	84.1	59.6	15.1	97.8
Dupuy	10.4	14	44	1	62	93.3	58.5	24.1	98.4
Eggers	14	110	87	18	145	85.9	62.5	55.8	89.0
Jacobs	14	80	150	15	339	84.2	69.3	34.8	95.8
Maisel	14	107	677	9	1134	92.2	62.6	13.6	99.2
Meune	14	13	21	0	23	100	52.3	38.2	100
Ricci	10	29	43	0	124	100	74.3	40.3	100
Sebbane	13.11	24	52	1	90	96.0	63.4	31.6	98.9
Thelin	14	67	207	3	201	95.7	49.3	24.5	98.5
Vafaie	10	27	44	1	59	96.4	57.3	38.0	98.3
Wildi	9	322	584	36	987	89.9	62.8	35.5	96.5

Abbreviations: TP = true positive; FP = false positive; FN = false negative; TN = true negative; NPV = negative predictive value; PPV = positive predictive value.
