# Supplementary material for: Diagnostic accuracy of adding copeptin to cardiac troponin for non-ST-elevation myocardial infarction: A systematic review and meta-analysis
Source: PLoS One. 2018 Jul 6;13(7):e0200379. doi: 10.1371/journal.pone.0200379 (PMC6034895; doi:10.1371/journal.pone.0200379)
Supplement: S4 Table — (PDF) [file pone.0200379.s004.pdf]

S4 Table. Patient characteristics of included studies

Study	Age (years)	Men (%)	Previous CAD (%)	Previous MI (%)	Previous PCI/CABG (%)	HTN (%)	DM (%)	HLD (%)	Stroke (%)	Smoking (%)	Family history (%)	Obesity (%)
Alquezar	69 (60-76)	67.0	33.7	NR	NR	67.0	28.6	NR	NR	NR	NR	NR
Bahrmann	81 ± 6	48.7	45.1	33.3	24.5/12.4	84.3	33.7	67.3	23.9	6.9	30.4	NR
Charpentier	58 ± 16	66.1	30.7	NR	NR	42.6	12.8	39.9	NR	28.9	NR	NR
Collinson	54 (44-64)	59.6	NR	5.8	3.8/0.9	35.4	8.1	23.6	NR	28.5	NR	NR
Dupuy	63 ± 17	63	NR	NR	NR	NR	NR	NR	NR	NR	NR	NR
Eggers	67 (58-76)	65.6	NR	37.5	17.5	42.8	18.3	38.3	NR	18.1	NR	NR
Jacobs	62 (51-73)	60.4	36.1	NR	NR	40.0	16.8	34.0	8.9	38.2	41.3	NR
Maisel	56 ± 13	56.8	38.5	27.9	29.7	69.8	28.8	55.9	33.1	10.0	45.3	NR
Meune	58 ± 14	63.8	NR	20.7	19.0	41.4	22.4	37.9	NR	32.8	34.5	32.8
Ricci	61 ± 16	64.3	31.1	21.4	15.3	60.9	19.4	30.1	NR	24.5	24.5	25.0
Sebbane	61 (48-75)	63.4	21.6	14.8	NR	34	14.1	35.1	10	36.6	NR	NR
Thelin	66 (55-76)	62.6	38.5	NR	21.1/12.3	53.6	20.7	38.7	10.9	14.4	NR	NR
Vafaie	64 ± 12	69.5	NR	20.6	38.2/11.5	73.3	23.1	60.0	10.7	20.0	44.9	35.1
Wildi	62 (50-75)	68.2	36.0	24.0	27.9	63.6	17.9	50.3	5.3	25.1	21.6	NR

*Continuous variables are presented as mean ± SD or median (interquartile range).

Abbreviations: CAD = coronary artery disease; MI = myocardial infarction; PCI = percutaneous coronary intervention; CABG = coronary artery bypass grafting; HTN = hypertension; DM = diabetes mellitus; HLD = hyperlipidemia; NR = not reported.
