# Supplementary material for: Diagnostic accuracy of adding copeptin to cardiac troponin for non-ST-elevation myocardial infarction: A systematic review and meta-analysis
Source: PLoS One. 2018 Jul 6;13(7):e0200379. doi: 10.1371/journal.pone.0200379 (PMC6034895; doi:10.1371/journal.pone.0200379)
Supplement: S5 Table — (PDF) [file pone.0200379.s005.pdf]

S5 Table. Sensitivity analysis for diagnostic accuracy of cardiac troponin alone and adding copeptin to cardiac troponin for non-ST-elevation myocardial infarction; except for one study that enrolled patients aged ≥ 70 years.

A. Cardiac troponin including cardiac troponin I and high-sensitivity cardiac troponin T

Diagnostic tests (95% CI)	cTn	cTn with copeptin	difference	*p-value*
Sensitivity	0.79 (0.73, 0.85)	0.91 (0.88, 0.94)	0.12 (0.10, 0.15)	< .001
Specificity	0.90 (0.83, 0.97)	0.60 (0.54, 0.66)	-0.30 (-0.33, -0.26)	< .001
PPV	0.61 (0.50, 0.73)	0.30 (0.19, 0.42)	-0.31 (-0.37, -0.24)	< .001
NPV	0.96 (0.95, 0.98)	0.98 (0.96, 0.99)	0.01 (0.01, 0.02)	0.001
AUC	0.91 (0.90, 0.90)	0.83 (0.81, 0.84)	NA	< .001

B. Diagnostic accuracy of adding copeptin to high-sensitivity cardiac troponin T

Type of cTn	cTnI				Hs-cTnT			
Diagnostic tests (95% CI)	cTnI	cTnI with copeptin	Difference	*p-value*	hs-cTnT	hs-cTnT with copeptin	Difference	*p-value*
Sensitivity	0.71 (0.60, 0.82)	0.89 (0.86, 0.93)	0.18 (0.13, 0.23)	< .001	0.83 (0.77, 0.90)	0.92 (0.87, 0.96)	0.09 (0.06, 0.12)	< .001
Specificity	0.96 (0.92, 1.00)	0.67 (0.62, 0.72)	-0.29 (-0.32, -0.26)	< .001	0.80 (0.67, 0.93)	0.54 (0.46, 0.61)	-0.26 (-0.31, -0.21)	< .001
PPV	0.73 (0.52, 0.95)	0.29 (0.14, 0.44)	-0.44 (-0.56, -0.32)	< .001	0.49 (0.40, 0.58)	0.44 (0.22, 0.65)	-0.05 (-0.13, 0.03)	0.101
NPV	0.96 (0.93, 0.99)	0.98 (0.96, 1.00)	0.02 (0, 0.04)	0.024	0.96 (0.94, 0.98)	0.93 (0.89, 0.98)	-0.03 (-0.05, -0.01)	0.001
AUC	0.93 (0.92, 0.95)	0.80 (0.78, 0.82)	NA	< .001	0.90 (0.88, 0.92)	0.81 (0.78, 0.83)	NA	< .001

Abbreviations: cTn = cardiac troponin; cTnI = cardiac troponin I; hs-cTnT = high sensitivity cardiac troponin T; 95% CI = 95% confident interval; PPV = positive predictive value; NPV = negative predictive value; AUC = area under the summary receiver operating characteristic curve; NA = not available.
