# Supplementary material for: Diagnostic accuracy of adding copeptin to cardiac troponin for non-ST-elevation myocardial infarction: A systematic review and meta-analysis
Source: PLoS One. 2018 Jul 6;13(7):e0200379. doi: 10.1371/journal.pone.0200379 (PMC6034895; doi:10.1371/journal.pone.0200379)

S1 Figure. Assessment risk of bias using Quality Assessment of Diagnostic Accuracy Studies - 2 tool

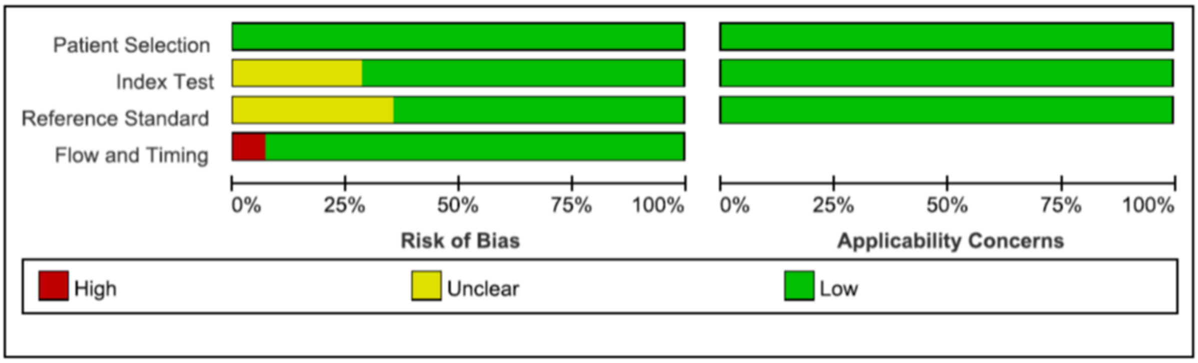

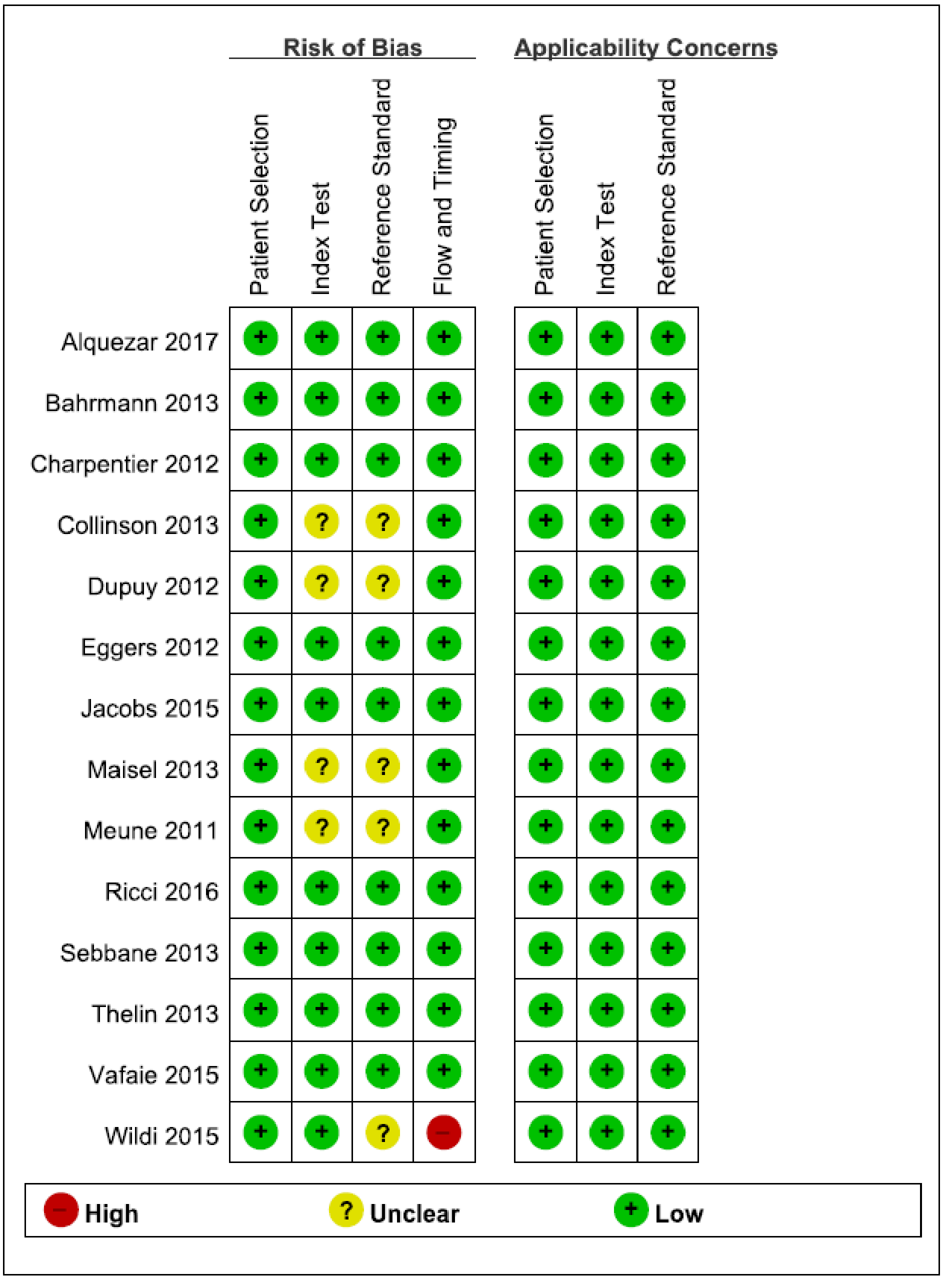

Supplement: S1 Fig — (PDF) [file pone.0200379.s006.pdf]
